# Applying deep learning and the ecological home range concept to document the spatial distribution of Atlantic salmon parr (*Salmo salar* L.) in experimental tanks

**DOI:** 10.1038/s41598-025-90118-9

**Published:** 2025-02-18

**Authors:** Santhosh K. Kumaran, Lars E. Solberg, David Izquierdo-Gomez, Hernan A. Cañon-Jones, Ingrid Mage, Chris Noble

**Affiliations:** 1 Nofima AS, Ås, Norway; 2 Nofima AS, Tromsø, Norway; 3 Universidad de Las Americas, Providencia, Chile

**Keywords:** Ecology, Computer science, Statistics, Behavioural methods, Imaging, Sensors and probes, Software

## Abstract

Measuring and monitoring fish welfare in aquaculture research relies on the use of outcome- (biotic) and input-based (e.g., abiotic) welfare indicators (WIs). Incorporating behavioural auditing into this toolbox can sometimes be challenging because sourcing quantitative data is often labour intensive and it can be a time-consuming process. Digitalization of this process via the use of computer vision and artificial intelligence can help automate and streamline the procedure, help gather continuous quantitative data and help process optimisation and assist in decision-making. The tool introduced in this study (1) adapts the DeepLabCut framework, based on computer vision and machine learning, to obtain pose estimation of Atlantic salmon parr under replicated experimental conditions, (2) quantifies the spatial distribution of the fish through a toolbox of metrics inspired by the ecological concepts home range and core area, and (3) applies it to inspect behavioural variability in and around feeding. This proof of concept study demonstrates the potential of our methodology for automating the analysis of fish behaviour in relation to home range and core area, including fish detection, spatial distribution and the variations within and between tanks. The impact of feeding on these patterns is also briefly outlined, using 5 days of experimental data as a demonstrative case study. This approach can provide stakeholders with valuable information on how the fish use their rearing environment in small-scale experimental settings and can be used for the further development of technologies for measuring and monitoring the behaviour of fish in research settings in future studies.

## Introduction

The measuring and monitoring of fish welfare in experimental settings is gaining increasing attention in both fundamental and applied fields of fish research^1–8^. This is an increasingly important topic, and research efforts aiming to document and understand the impacts of experimental conditions upon the welfare of these animals are ongoing. Animal welfare in EU research settings is overseen by the European Directive 2010/63/EU on the protection of animals used for scientific purposes^9^, which is central to the delivery of the Reduction and Refinement goals of the 3R framework proposed by^10^.

The need for developing integrated welfare indicator toolboxes for assessing fish welfare in laboratory and aquaculture settings has been highlighted, as well as the need for common methods for documenting, auditing, and improving fish welfare under scientific studies^2,4^. This is a complex task, and the need of a multidisciplinary approach and the use of non-invasive methods has been proposed^5,6^. And which is central to Directive 2010/63/EU.

Welfare Indicators (WIs) are the tools a stakeholder uses for monitoring fish welfare. They can either be Operational Welfare Indicators (OWIs) that are simple to use and implement in applied settings, or Laboratory-based Welfare Indicators (LABWIs) that are more complex and need further analysis, often in specialist settings or situations. They can also be classified as outcome-based (direct or animal-based indicators), focusing on indicators measured directly on the animal at the group or individual level, or input-based (indirect or environmental-based indicators), focusing on the resources the animal is subjected to. Each indicator must be intrinsically linked to one or more welfare needs and there is currently no single indicator that addresses all welfare needs, meaning an array of tools must be used in a welfare audit or monitoring programme.

Behavioural indicators have high utility for documenting and auditing animal welfare^11^, in a range of animals including fish^12–15^. Behavioural indicators can also be used to reduce the impact of welfare risks and thus improve welfare^16^. The monitoring of various behavioural indicators such as fish distribution, aggression, activity/swimming levels and behaviours that can be indicative of potential negative welfare states and can help a stakeholder understand how fish respond to their environment and identify potential stressors^17^. There is also an increasing focus on how behaviour may reflect positive welfare states and the fulfilment of fish’s welfare needs, e.g.,^18^. However, fish behaviour is often difficult to quantify, and the auditing process is usually manual and time-consuming^19^. This limits its utility and application in a range of settings including in experimental studies.

Home range is an ecological behavioural metric that is defined as the spatial area occupied by an individual during its day-to-day activities, excluding any atypical excursions^20^. Home range is often defined as the area where the individual spends 95% of their time and this can be further broken down into indices such as the core area, which is the area where an individual spends 50% of its time^21,22^. Home range and core area data often consist of individual positional coordinates visualised as a map, smoothed via Kernel Density Estimation (KDE), which then delineates home range and core areas as closed contours over the map^21^. Metrics of home range have been used in ecological and applied research on fish such as assessing the influence of marine fish cages on wild populations of fish (for a review see^23^), as well as to document the behaviour of escaped fish^24,25^. The spatial distribution, orientation, and social interactions of fish can be driven by e.g., environmental factors such as variability in dissolved oxygen saturations. Water temperature, water velocity, and feed availability (e.g.^26^). Spatial distribution metrics are relevant for examining processes such as acclimation, habituation, and learning, which are critical for understanding how fish adapt to their rearing environment^13^. Monitoring the variability in spatial distribution therefore has potential utility in aquaculture research, especially those that involve small numbers of fish in small-scale experimental settings. For example, numerous experimental studies do involve small numbers of animals within a rearing system, such as those studying fish health^27^ and also in general behavioural studies^28–30^. Home range and core area metrics can provide valuable insights into how fish are affected by and interact with their surroundings^13^. Reduced home range size may indicate stress, disease, or challenges with the rearing system design and operation, while increased core area usage can also suggest the presence of stressors^31,32^. Analysing spatial overlap between fish groups can reveal social interactions and avoidance behaviours^28^. They can also indicate areas that the animals have preference for within a rearing system.

The emerging use of digitisation in fish welfare monitoring can provide automated tools for quantifying fish behaviour in experimental and commercial settings. This can provide stakeholders with a wider and more rapid overview of changes in fish behaviour, helping them make more informed and robust decisions in both the short- and longer-term. Computer vision (CV) plays an increasingly key role in the development of Artificial Intelligence (AI) in various domains such as transport^33^, healthcare^34^, manufacturing^35^, agriculture^36^, and cyber security^37^. There is an increasing focus on its potential application in fish research^38^ and aquaculture^39,40^. A developing trend in fundamental and applied fish research includes the application of AI, such as machine learning algorithms, for mining high-dimensional features and depth information extracted from videos to understand feeding^41^ and grouping behaviour patterns^42^. It has been used to e.g. measure and monitor swimming behaviour^43^ and measure feeding activity^44^. Studies have also focused on using AI to identify stressful states in fish and potential stressful situations that can put fish welfare at risk^16^. The relevance and utility of using deep learning in these settings is increasingly recognized by the research community^45–48^.

Emerging technologies in behaviour monitoring within aquaculture show great promise as tools for gathering information that can contribute to enhanced production and pave the way for improved efficiency and sustainability^49^. Vision-based sensors, acoustic-based sensors, and biosensors are now widely used for fish tracking, counting, and behaviour analysis^50^. These technologies leverage computer vision, hydroacoustic methods, and physiological data to provide comprehensive insights into fish health and behaviour. However, current behavioural monitoring methods face several challenges that highlight the need for automation. Manual observations are time-consuming, labour-intensive, and prone to human error, leading to inconsistent and subjective data^49^. Additionally, these methods often lack real-time data, resulting in delayed responses and missed early signs of stress or disease^50^. They can also be invasive, causing stress and potential harm to the fish, and frequent human interaction can disrupt a fish’s behaviour. Environmental factors such as poor visibility and dynamic conditions further complicate consistent monitoring^51^. Automation addresses these issues by providing accurate, consistent, and objective data through potentially continuous, 24/7 monitoring^49,50,52,53^. Automated systems can deliver real-time alerts for abnormal behaviours, enabling prompt intervention and proactive management, which can then be used to improve fish health and welfare. Moreover, non-invasive automated methods reduce potential stress and harm to the fish, allowing for natural behaviour observation. Advanced sensors in automated systems can adapt to various environmental conditions, offering a comprehensive understanding of fish behaviour and environmental interactions. These benefits make a strong case for the adoption of automated behaviour monitoring in aquaculture research. However, the use of AI in this field is still in its early stages, with most applications are currently at the proof-of-concept or developmental phase.

This study introduces a novel approach that combines deep learning techniques with home range analysis to automatically extract and analyse the spatial distribution of small numbers of Atlantic salmon (*Salmo salar* L.) parr in experimental tanks. We employed the deep learning framework DeepLabCut^46^ to identify key body parts of the fish and used this information to determine the distribution of fish within the tank via the application of the ecological home range concept. We then quantified a set of metrics characterising the home range of the fish before finally, in a small-scale case study, investigating if these metrics are able to capture differences in behaviour around feeding times. Our current approach focused on collective behaviour, which provides insights into group-level distribution patterns. We believe that this approach is well-suited for increasing our understanding of the dynamics of fish populations in experimental and aquaculture research settings. Additionally, these metrics can potentially help monitor the impact of external factors, such as water quality and feeding regimes, on fish behaviour and welfare. Unlike simple image-based analysis, the tool has the potential to incorporate fish orientation and movement data, which are critical for studies on schooling behaviour, social interactions, and potential movement patterns. These features allow for advanced investigations into fish behaviours such as alignment, cohesion, and polarization, which are essential metrics in understanding group dynamics and welfare.

## Methods

### Experimental design and set-up

**Fig. 1:**
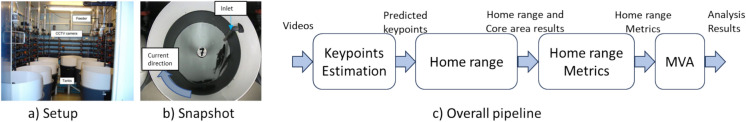
(**a**) An overview of the experimental setup used in the original experiments^28^ (**b**) A snapshot of a video frame from the over water camera. (**c**) Keypoints are estimated from the videos and home range is estimated from the keypoints. Feature metrics corresponding to the home range are calculated from the home range and finally, multivariate analysis (MVA) is performed on the home range metrics.

The study reuses video footage collected automatically from a previous experiment conducted at the Aquaculture Research Station in Tromsø on Atlantic salmon parr, see^28^ for details. In brief, six fish weighing 137 g (mean wet weight) were transferred to each of three tanks (freshwater flow through, 300 L tanks) and allowed to acclimate for 14 days. Fish were fed to satiation over a 30 min period each day at ca 1.5% body weight/day at 10:00 h using pre-programmed automatic feeders. This meant there was no staff disturbance both during and around feeding times. The feed (pellets) was dispersed at the surface over a limited area across all tanks. Feed was delivered in intervals during the 30 min period and our analysis focused exclusively on the first feeding interval during this time. This approach ensured consistency in observations and targeted the fish’s initial behavioural responses to feed delivery. Water temperature was maintained at 10 ± 2 °C during the experiment. The tank setup and a video frame snapshot are shown in Fig. 1a,b. DeepLabCut (DLC) was used for behavioural analysis by identifying key body parts of the fish (keypoints). An overview of the pipeline used in the study is shown in Fig. 1c.

### Dataset

The dataset used in our experiments consists of 1-min video recordings capturing three distinct phases: (1) 10 min before feeding (pre-feeding), (2) the first minute of feeding, and (3) 1 h after feeding ceased (post-feeding). As the recordings were limited in duration and did not address the whole period before, during and after feeding, we only have the opportunity to see if the pipeline can detect behavioural differences in certain time periods in and around feeding, and it cannot be used to detect e.g., food anticipatory behaviour in this study. Video recordings were made using Panasonic© VWR42 cameras with Panasonic© WV-LA4R5C3B lenses located 1 m above each tank. The videos were recorded at 25 frames per second (fps). We have used data from three tanks (Tank-3, Tank-7, and Tank-8) to audit variability in home range and core area of the fish groups in and around feeding. We analysed the videos for the first five days after tank acclimation (Days 14–18). There was no staff disturbance of the fish during filming periods or around these mealtimes.

### Keypoints estimation

**Fig. 2:**
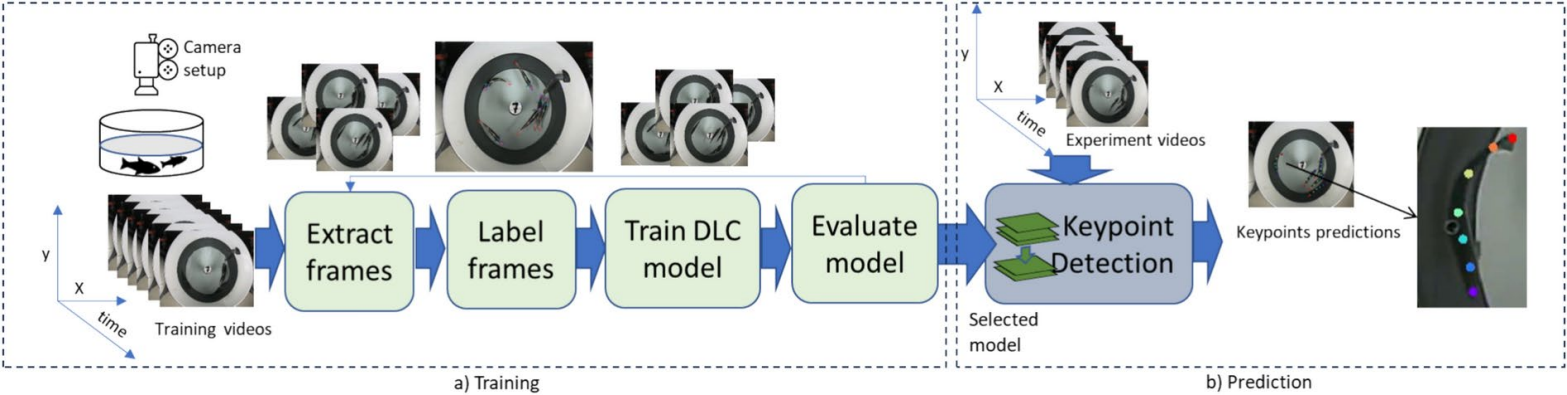
Showing an overview of the training and testing of the proposed framework for keypoint detection. The selected model is then used for the prediction of keypoints on the experiment videos.

We developed a deep learning model to estimate several keypoints on each fish using DLC^46^ which uses a ResNet50 architecture as the backbone^54^ as depicted in Fig. 2a,b. We employed 674 annotated frames for training and 36 for testing. Each fish was annotated with seven keypoints corresponding to the snout, rear of the head, anterior edge of the dorsal fin, posterior edge of the dorsal fin, the adipose fin, caudal fin peduncle and the posterior edge of the caudal fin as shown in Fig. 2b. The training and testing of the model was carried out using a workstation (AMD Ryzen Threadripper 3970X 32-Core Processor 3.69 GHz) with two GPUs (NVIDIA GeForce RTX 3090).

### Home range estimation

1 $$R\left( \gamma \right) = \{ \left( {x,y} \right)|p\left( {x,y} \right) \ge \gamma \} , \;\;{\text{where}}\;\;\mathop \int \nolimits_{R} p\left( {x,y} \right)dA = \tau$$

**Fig. 3:**
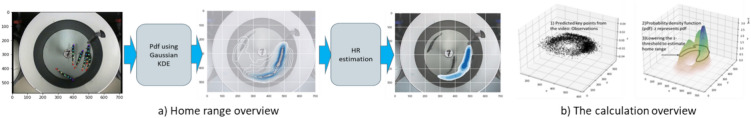
(**a**) 3D illustration of the probability density function calculation and the home range estimation. z-axis represents the probability density function. Core area corresponds to the regions ${\text{R}}$ given by Eq. (1) with ${\uptau } = 0.5$. (**b**) Home range estimation pipeline.

The trained model was used to predict ${\text{N}}$ keypoints $({\text{x}}_{{\text{i}}} ,{\text{ y}}_{{\text{i}}} )$ with a probability confidence above 0.6, on test/experimental videos, where ${\text{i}} = 1 \cdots {\text{N}}$. The pixel positions (data) on the video were used to estimate the probability density function of fish distribution using the Gaussian Kernel Density Estimate (KDE)^21^. The home range and core area correspond to the region $R$ as given in (1) for a specified threshold $(\tau )$ and where $\gamma$ solves the integral equation. We have considered threshold values of ($\tau = 0.95$) and ($\tau = 0.5$), for the home range and core area respectively, corresponding to where fish spend 95% and 50% of their time during each auditing period, respectively^22^. An illustration of the estimation of home range and core area is shown in Fig. 3.

We chose an iterative approach to solve the integral Eq. (1) where we successively include areas in the probability distribution of decreasing density until the sum probability of the region reaches $\tau$. The solution for $R$. May correspond to multiple connected areas and may correspond to different groupings of fish in different regions.

The analysis of the one-minute videos was split into 10 s contiguous time windows to provide replicates for the multivariate analysis and also to detect potential acute changes in the distribution of the fish.

#### Home range metrics

**Fig. 4:**
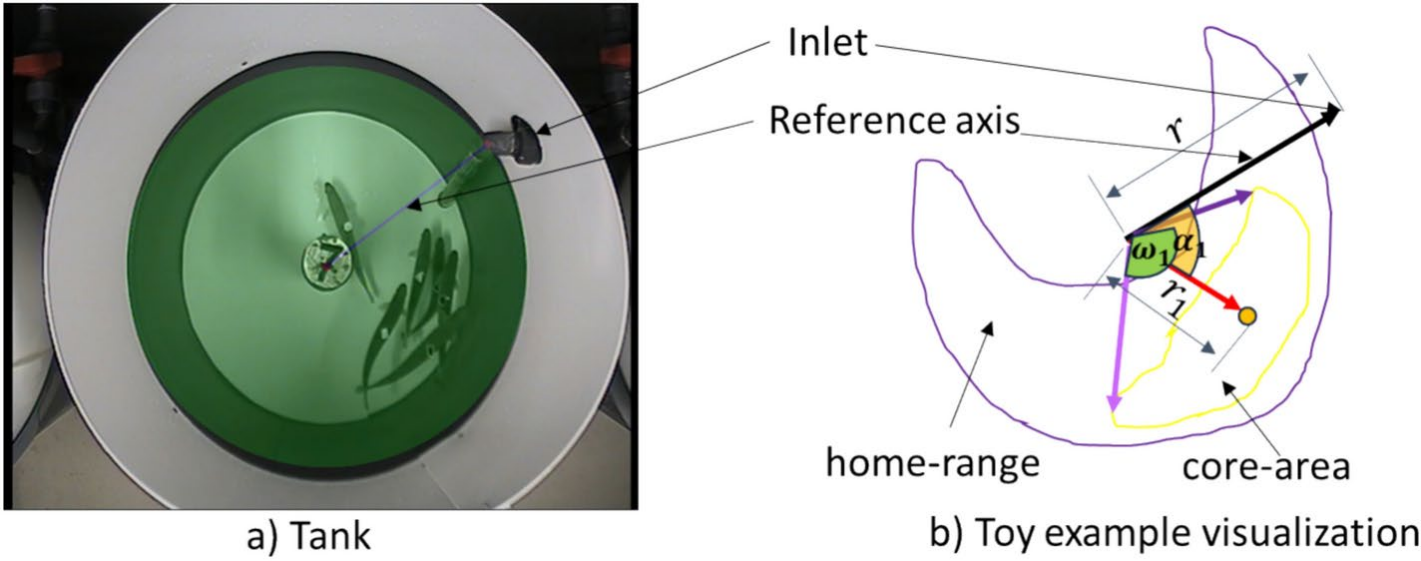
An illustrative visualization of the metrics derived from home range and core area. (**a**) An example where the masked region, which is set manually, represents the area considered for the metrics’ calculations. (**b**) A visualisation of the metrics. The home range corresponds to the area marked by the purple contour and the core area corresponds to the region represented by the yellow contour.

We characterized home range and core area in terms of metrics outlined in (Table 1). Additionally, Fig. 4 gives an insight into the relevant features described in the table.

**Table 1:** Outlining selected home range metrics and their definitions.

Home range metric	Formula	Description
*rel-core-area*	$\frac{core - area}{{\pi r^{2} }}$	A relative measure of core area in relation to the total tank area, to better understand the magnitude of preferred regions for fish within the space available
*rel-home-range*	$\frac{home - range}{{\pi r^{2} }}$	A relative measure of the home range in relation to the total tank area, to better understand the magnitude of fish dispersion within the tank
*core-ratio*	$\frac{core - area}{{home - range}}$	The ratio between the core area and home range. It provides insight into the preferred areas within the home range
*Groups*	The number of core area clusters ($K$)	This metric assesses whether fish aggregate in one or several clusters within the tank
*centre (x,y)*	$\frac{{\mathop \sum \nolimits_{{{[}I = 1{\text{\} }}}}^{k} w_{i} \cdot (x_{i} ,y_{i} )}}{{\mathop \sum \nolimits_{{\left\{ {i = 1} \right\}}}^{{\left\{ K \right\}}} w_{i} }}$	The positioning the centroid of the fish as a group within the tank. The mean of core area centroids $\left( {x_{i} , y_{i} } \right)$ of weight ($w_{i}$) proportional to their core area is calculated when several fish groups ($K$) are present. It helps in determining the fish distribution as a group
Water Inlet Preference (*WIP*)	$\alpha$	The angle between the two radii formed between the centre of the tank, the water inlet and the centre of the core area or mean core areas of the fish (if several). It helps to determine fish distance to the water inlet
Angular Distance of the Core area (*ADCA*)	$\cup \omega_{i}$	The angular span of the core area measured in radians (or the sum of them when there are several fish groups). It represents the dispersion of fish within the tank
*Circularity*	$\frac{4\pi core - area}{{core - area - perimenter^{2} }}$	A measure of the shape of the core area. It is to help document whether fish are swimming in an aligned formation (ellipse form) or swimming in a circular formation
Core Area Centrality (*CAC*)	$\frac{{\frac{{\mathop \sum \nolimits_{{{[}I = 1{\text{\} }}}}^{k} w_{i} r_{i} }}{{\mathop \sum \nolimits_{{\left\{ {i = 1} \right\}}}^{{\left\{ K \right\}}} w_{i} }}i}}{r}$	The ratio of the distance ($r_{i}$) of the centroid from the centre to the radius of the tank (r). to discriminate whether fish prefer to swim closer to the tank walls (thigmotaxis) or in central areas of the tank. The mean centroid is relevant when several groups ($K$) were documented

### Multivariate analysis

Since the home range and core area proxies are represented as images, it is more natural to use the derived numerical metrics (Table 1) for statistical analyses and for exploring their relationships with other experimental data. In this study, cluster analysis and multivariate analysis of variance was employed to evaluate the associations between the metrics and experimental factors, guiding how these metrics can be analysed in this and future studies.

#### Relationships between metrics

The redundancy between metrics was examined by a classical Pearsson correlation coefficient, which is suitable for characterising linear dependencies. In addition, the novel ϕ_K_ correlation coefficient was calculated to capture nonlinearities between the metrics^55^. Hierarchical clustering of the correlation matrix was performed using Ward’s linkage method, to identify groups of related metrics. A total of 270 video segments of 10 s duration were used to assess the relationships between metrics.

#### Ability of metrics to distinguish between behaviours

We investigated how the different metrics were associated with feed delivery across limited numbers of tanks (n = 3) and days (n = 5) using ANOVA-Simultaneous Component Analysis (ASCA). ASCA is a multivariate version of the analysis of variance (ANOVA), suited for analysing multiple collinear variables stemming from an experimental design^56,57^. The advantage of ASCA is to provide estimates of multivariate effect sizes with corresponding p-values, in addition to PCA-based visualisation of each effect. Confidence ellipsoids for factor level comparisons were estimated as described in^58^. In this work, the variables represent the mean and deviation of each metric (Table 1) during one-minute video segments taken before, during and after feeding, in three tanks, for five successive days. We used the same time period for non-feeding for consistency in comparison. The factors in our ANOVA model were therefor “Feeding”, “Tank”, and “Day”, and we included all two-level interactions.

## Results

### Keypoint estimation

**Fig. 5:**
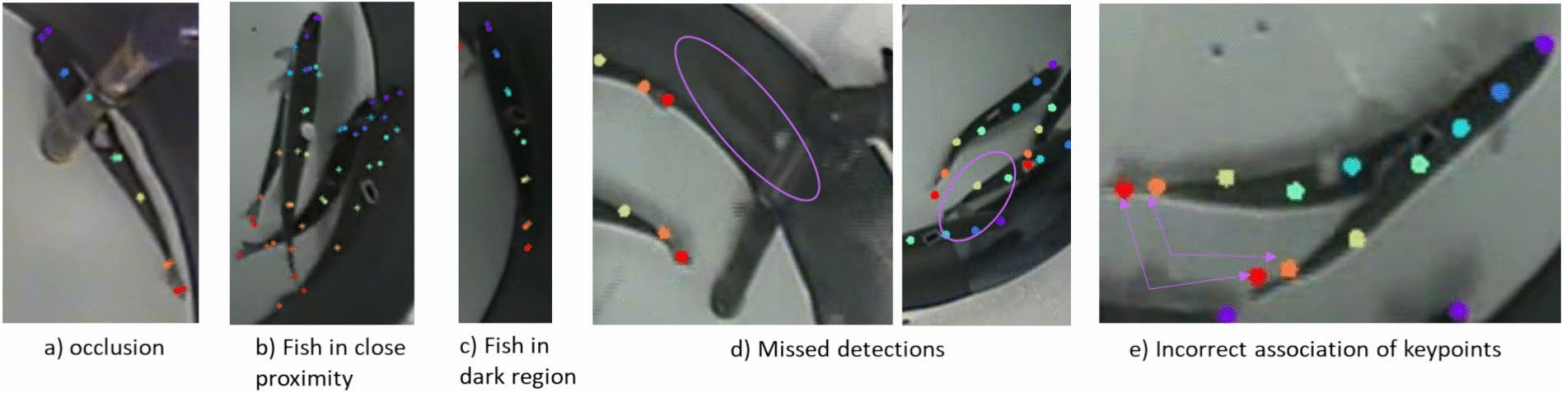
Samples from the test frames. [+] indicates the ground truth and [.] the predictions. (**a**) Anterior edge of the dorsal fin prediction in occluded region (**b**) Predictions when fish are in proximity (**c**) prediction in a dark region of the tank. (**d**) Some of the pitfalls shown in the form of missed detections (**e**) Association of keypoints to the wrong fish between continuous frames.

The keypoint detection accuracy, measured as the ratio of correct predictions to total predictions, was 0.92 ± 0.02 (Table 2a). The model’s training and test errors were both less than 0.04 (Table 2b). Examples of predictions on test frames are shown in Fig. 5.

**Table 2:** (a) Prediction accuracy and (b) model prediction error.

(a)		
Body parts	Accuracy	
Snout	0.92	
Rear end of head	0.92	
Anterior edge of dorsal fin	0.93	
Posterior edge of dorsal fin	0.96	
Adipose fin	0.94	
Caudal fin peduncle	0.88	
Posterior edge of caudal fin	0.89	

(b)		
Parameter	# Samples	Error (as proportion of body length)
Training	674	0.02
Test	36	0.04

### Home range and core area estimation

**Fig. 6:**
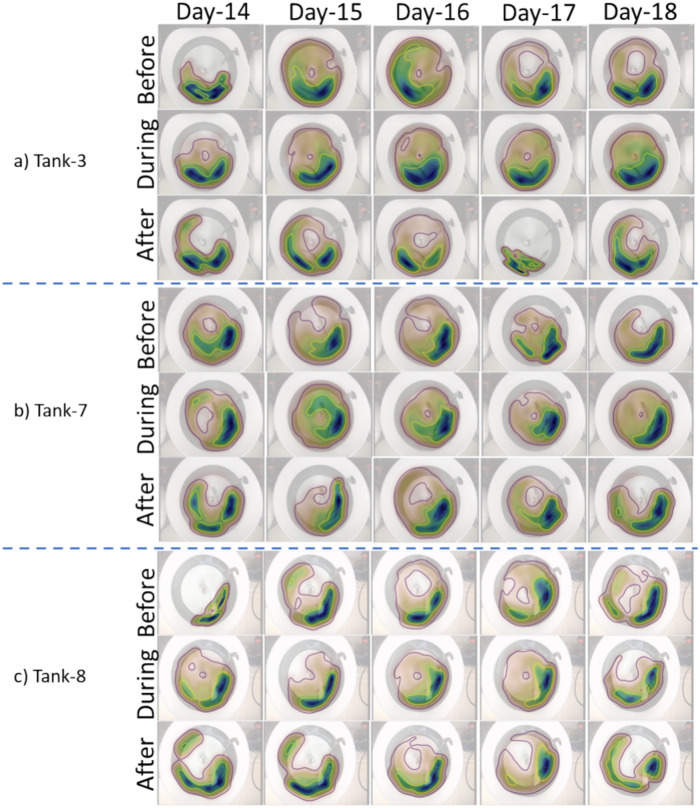
Daily overview of the home range and core area over the 5-day monitoring period in 3 tanks, illustrating differences in the spatial distribution of fish before, during and after feeding (1 min of observations in each period). The visualizations in each tank are organized into three rows, each corresponding to a specific phase: before, during and after feeding. The columns represent different days, from day 14 to day 18. All the tanks have same design as illustrated in Fig. 4.

Figure 6 outlines snapshots of the home range and core area for the three different tanks before, during and after feeding on different days. It appears that the home range and core area exhibit changes within and between different feeding periods, days, and tanks. In the subsequent sections, we explore home range and core area variability in terms of the defined metrics.

**Fig.7:**
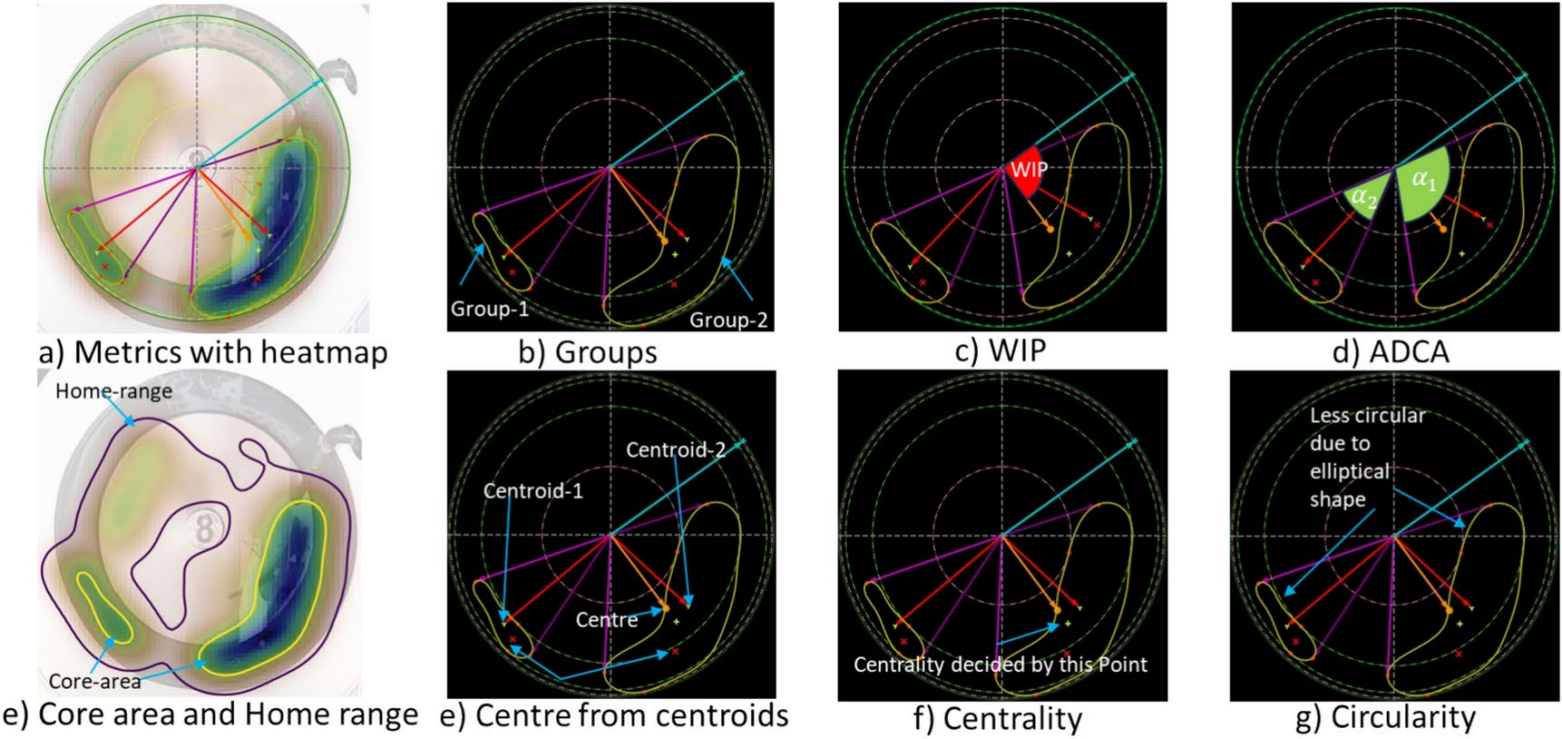
Detailed illustration of the various metrics for Tank-8, before feeding on day-18. The illustrations with the black background are to highlight the estimated metrics. Concentric dashed circles (green for group 1, pink for group 2 as in (b)) indicate the radial extent of core areas.

The metrics extracted from Fig. 6 are also summarised in Table 3. A key metric describes the fish’s preference for spatially distributing themselves ca. 90° downstream from the water inlet. The home range metrics are also visualised for a single tank (Tank-8) during a single pre-feeding period (day-18) in Fig. 7.

**Table 3:** The average home range and core area metrics**.**

Category	Identifier	Groups	Centre(x, y)	ADCA (radians)	Circularity	WIP (radians)	CAC	Core-ratio	Rel-core-area	Rel-home-range
Tank	Tank-3	1.78	(314.55, 424.71)	2.77	0.47	2.23	0.58	0.29	0.16	0.57
	Tank-7	1.85	(432.58, 364.52)	2.93	0.51	1.53	0.53	0.28	0.15	0.55
	Tank-8	2.19	(371.68, 368.02)	2.58	0.51	1.67	0.63	0.27	0.13	0.49
Day	Day-14	2.26	(369.95, 397.07)	2.70	0.47	1.81	0.60	0.29	0.14	0.48
	Day-15	1.88	(372.59, 385.28)	2.82	0.48	1.85	0.58	0.28	0.15	0.55
	Day-16	1.67	(367.15, 384.92)	2.85	0.51	1.95	0.56	0.27	0.17	0.63
	Day-17	1.92	(387.80, 377.10)	2.53	0.52	1.56	0.59	0.28	0.14	0.50
	Day-18	1.97	(367.18, 384.38)	2.88	0.49	1.85	0.58	0.28	0.15	0.53
Relationship with feeding	Pre-feeding	1.95	(375.58, 390.21)	2.75	0.50	1.81	0.59	0.28	0.15	0.55
	Feeding	1.71	(384.51, 375.67)	2.81	0.50	1.69	0.55	0.28	0.15	0.54
	Post-feeding	2.15	(358.72, 391.37)	2.71	0.48	1.92	0.61	0.28	0.14	0.51

### Multivariate analysis

**Fig. 8:**
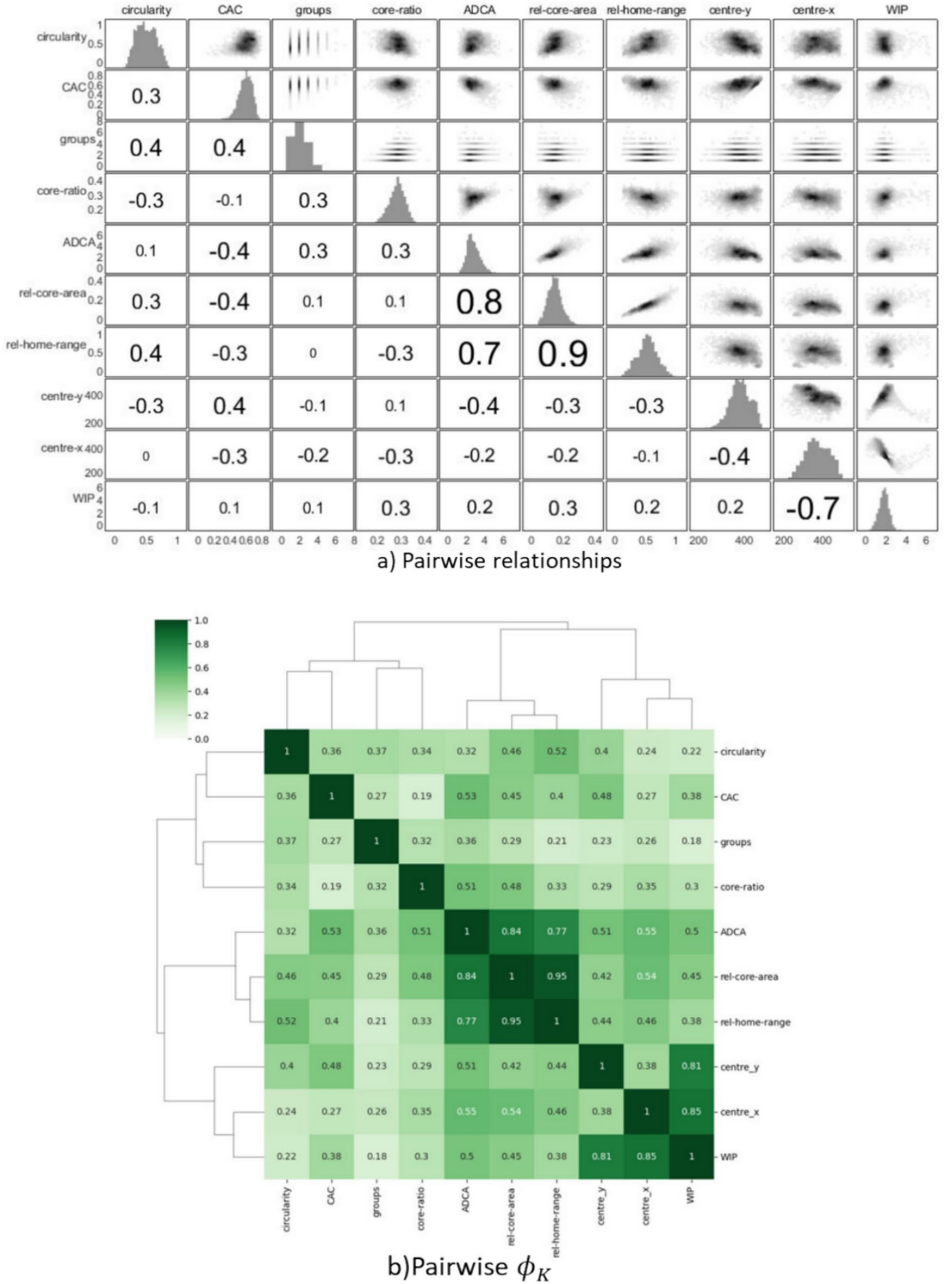
Depiction of pairwise relationships between different metrics a) Pairwise relationships between variables. The distribution of each metric is presented along the diagonal. Density scatter plots are presented above the diagonal, while the corresponding Pearson correlation coefficients are presented below the diagonal. b) Pairwise $\phi_{{\text{K}}}$ correlation coefficients, clustered by hierarchical clustering. The $\phi_{{\text{K}}}$ takes values between zero and one and can be used to measure the strength of both linear and non-linear relationships.

**Fig. 9:**
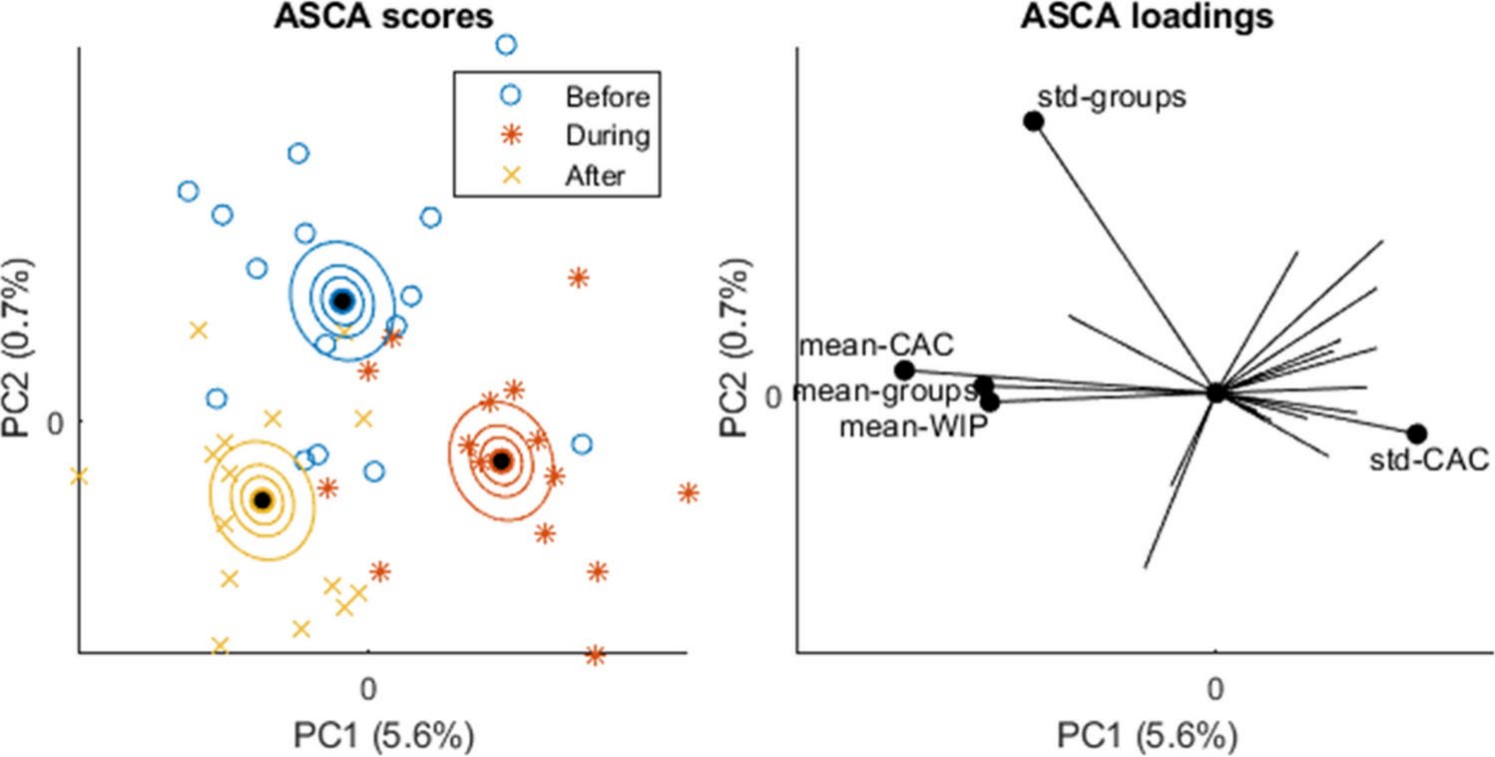
ASCA scores and loadings for the main effect of feeding, i.e. corrected for variation between days and tanks. The scores illustrate differences between feeding events. Each marker in the score plot represents a video taken at a certain time, in a certain tank, on a certain day. The black dots represent group means, and the ellipsoids represent confidence regions of the group means. The loadings show which variables that span the differences between groups. For clarity, only the most influential loadings (largest absolute values) are marked.

Figure 8 depicts the pairwise analysis of different features used in multivariate analysis. The most coherent group was formed by *Rel-core-area*, *Rel-home-range* and *ADCA.* These three metrics had a high degree of linear correlation and were therefore redundant. Another group was formed by *Centre-x*, *Centre-y* and *WIP*. *Centre-x* and *Centre-y* had sinusoidal relations to *WIP* (Fig. 9a), resulting in strong ϕ_K_ correlation coefficients. *Circularity*, *CAC, Core-ratio and Groups* metrics showed weak correlations to the other metrics and therefore provided unique information. This meant that a subset of six metrics (e.g. *Rel-core-*area, *Centre*-x, *Centre*-y, Circularity, CAC, Core-ratio and Groups) are enough to fully characterise aspects of the home range and core area in this dataset. In spite of the redundancy present in the set of metrics, all metrics were used in the multivariate analysis as the redundancy is automatically handled and to aid the interpretation of the results. Multivariate analysis also offers valuable insights into the relationships among all metric features.

Multivariate ANOVA splits the total variability into independent parts caused by the factors *Feeding*, *Tank* and *Day*. Effect sizes and p-values are given in Table 4. The table showed that tank as a factor had the largest effect (effect 14.4; *p* < 0.001) followed by day (effect 11.6; *p* = 0.07) and feeding (effect 6.4; *p* = 0.07). None of the interactions were even close to significant (*p* value in range 0.23–0.71). Figure 9 shows ASCA scores and loadings for the main effect of *Feeding*. The scores plot illustrates the systematic differences between before—during—and after feeding. All the group averages were significantly different since the confidence ellipsoids of the group means did not overlap. However, most of the variation was explained by the first principal component, meaning that it was mainly the “during” feeding behaviour that differs from “before” and “after”. The loadings showed which of the metrics were responsible for these differences; *WIP*, *CAC* and *groups* had higher mean values before/after than during feeding, while the variability in CAC was higher during feeding. This could be attributed to the movement of fish during feeding causing the home range and core area to change.

**Table 4:** Effects from the multivariate ANOVA.

Source of variation	Degrees of freedom	Effect size (%)	*p* value
Feeding	2	6.4	0.07
Tank	2	14.4	< 0.001
Day	4	11.6	0.07
Feeding × Tank	4	6.0	0.71
Feeding × Day	8	14.3	0.56
Tank × Day	8	17.7	0.23
Residuals	16	29.6	

The effect sizes are given as a percentage of the explained variance. *p* values are estimated by permutation tests.

The ASCA scores and loading plots for the effect of *Tank* (not shown) revealed that there were significant differences between all three tanks, specifically related to WIP, centroid-x, centroid-y, and CAC. This might indicate that external, tank-specific factors affect the preference for certain regions in the tank. The variation between days (not shown) seemed more random and could not be attributed to any specific metrics.

## Discussion

This article presents the combination of two approaches for monitoring the behaviour of small groups of fish in small scale laboratory tanks. We deliberately selected short observation periods for each tank within a day (1 min before, during, and after feeding, totalling 3 min) and limited the study to a five-day timeframe as our primary goal was to evaluate the DLC tool for measuring home range and core area metrics, using the source data as a case study. We then further explored potential changes in fish distribution around feeding times and across different tanks. Any potential behavioural insights should therefore be interpreted with this limited time frame in mind.

Our study uses an existing deep learning-based key-point detection software toolbox, namely DeepLabCut, to estimate keypoints on individual fish. The keypoints are then used to document the spatial distribution of the fish and its variability using a toolbox consisting of various metrics related to spatial distribution in relation to the ecological concepts of home range and core area. To our knowledge, this is the first time these proposed metrics have been applied in an aquacultural research setting using salmonid fish. As a proof of concept, the aforementioned framework was applied to document and analyse potential differences in fish behaviour between feeding and non-feeding periods, across different tanks and over different days.

The existing key-point estimation software tool, DeepLabCut, which has been used to study animal behaviour in previous studies^59,60^, proved successful in monitoring the distribution of Atlantic salmon parr within the replicated experimental design presented in the current case study. The continuous estimation of all the keypoints obtained over time allowed us to visualise the spatio-temporal distribution of fish in each tank. This distribution was then examined using home range and core area concepts.

### Keypoint estimation

The keypoint detection accuracy was greater than 0.9 for all keypoints apart from those associated with the caudal fin. The relatively lower prediction accuracy for the caudal fin region can be attributed to difficulties in locating the caudal fin due to its movement; this could be improved with higher-resolution cameras or with videos recorded at a higher frame rate. Prediction accuracy did decline when water surface reflections were present, although this is a challenging scenario even for manual auditing. With individual keypoint detection rates at approximately 90% and with seven keypoints per fish, it is very likely that almost all fish are detected almost all the time. It can be noted that the home range and core area metrics are relatively resistant to limitations in keypoint detection, as they are based on the collective information from all detected keypoints. Even if some keypoints are not detected accurately, the overall spatial distribution of the fish can still be reliably estimated. Another advantage of using keypoints instead of bounding box-based detection to represent each fish is the enhanced opportunities for monitoring their localisation and space use, which provides the possibility of applying metrics related to home range and core area in this study. This is further strengthened by the low location error of these points, at a small fraction of fish length. Beyond spatial distribution analysis, the detection of individual fish key points enables the study of additional behavioural phenomena. For instance, fish orientation could be analysed to explore changes in rheotaxis during smoltification, anti-parasite behaviours, or the degree of polarization within groups (e.g., shoaling or schooling)^61–66^. Similarly, inter-fish distances could be used to investigate social interactions and group dynamics, offering valuable insights into individual and group based welfare indicators^28^. These aspects represent promising directions for future studies utilizing this methodology.

Two known issues with detections of multiple objects in videos with occlusions are (1) the association of keypoints to fish, and (2) the tracking of these keypoints over time, thus limiting the capabilities for exploring spatio-temporal characteristics. A consequence of this was that higher prediction errors occurred when fish were in close proximity, likely due to occlusions. However, these issues are circumvented when considering the home range and core area of the entire group of fish as the correct association of keypoints to specific fish is irrelevant in so far as fish distribution is basically equal to the distribution of body parts. The stability of the region boundaries is low for home range as opposed to that of core area because there are many more points outside the region contributing to the defined boundary. This supports the selection of core area as the basis for further description in terms of the chosen metrics, some of which are sensitive to the region boundaries.

Interpreting the home range and core area metrics: The visualisation of the home range and core area preferences gives an insight into the variability in the spatial distribution of fish over short time scales within and between tanks and within and between days. While the results we present illustrates home range and core area metrics for 1-min durations during pre-feeding, feeding, and post-feeding events, the tool has the potential to monitor these metrics continuously over any desired time resolution. This capability makes it a valuable resource for real-time monitoring applications. The choice of a short temporal resolution period also influences the granularity of home range variability and adds to the utility of our findings. It is evident that fish have preferred areas in the tanks within our auditing periods. However, this preference can change between days in the same tank and also differs between tanks. It was also noted that home range was variable and can extend over wider areas, which may be related to varying biotic or abiotic factors (e.g. variability in behavioural cues within the group, or tank disturbance) on any given day^67^. Breaking down the one minute video clips into 10 s windows helped detect and monitor rapid changes in the spatial distribution of the fish.

One of the key findings is that fish preferred an area around roughly 90⁰ downstream from the water inlet pipe (WIP in range 88°–128° in Table 3), the area where water flow was directed into the tank from the water inlet. This preference may be associated with water quality parameters or fluid dynamics in the tank, but as our observations were for short time periods and more importantly, flow dynamics and water quality were not profiled, we do not wish to draw further conclusions on the potential drivers for this. However, we do wish to draw the reader’s attention to this finding for consideration in potential future studies, and the tools utility in detecting this.

Another stable metric is *rel-core-area* (or similarly *core-ratio*) with the fish occupying only 13–17% of the tank, 50% of the time. This reflects the tendency of fish to swim closely to other fish rather than distributing evenly through the tank.

The ASCA analysis showed that Tank was the factor that had the largest effect, with multiple drivers including primarily *centre-x*, *centre-y*, *WIP* and *CAC*. When considering Tank 8 as an example, the visual impression of elongated core areas along tank walls finds support in higher *CAC* and larger *groups*, the latter making the higher *CAC* more significant because the average centre of multiple groups leads to lower *CAC* values. When Day and Tank effects were corrected for, the ASCA analysis showed a significant difference between before and after versus during feeding, with key drivers including mean-group, mean-*WIP*, mean-*CAC*, std-*Groups* and std-*CAC*. Outside of feeding times, fish positioned themselves closer to the tank walls (higher mean-*CAC* values), with fish tending to split up in multiple groups (higher mean-Groups). The latter may be explained by fish swimming in a stationary manner which would derive in several zones of high-density estimations and depicted as several core areas within the home range (see examples in Fig. 6). Whilst a preference for the tank periphery may be indicative of thigmotaxis, or an avoidance of open areas due to a potential negative welfare state (e.g^68^), as the tank inlet was also in close proximity to the tank wall, and the fish exhibited a preference to be downstream of it, we do not consider these spatial preferences to be negative in this current study.

During feeding, the lower mean-Groups (Fig. 9, ASCA loadings) may be a product of foraging behaviour, such as the fish dispersing and chasing pellets, which led to a more uniform distribution in the tank, which is then interpreted as a single group. In a similar vein, these movements may lead to higher variability in certain metrics such as CAC, which exhibits a higher std-CAC value (Fig. 9, ASCA loadings). In general, it seems reasonable that a more stable fish distribution implies lower variability in metrics, and conversely higher variability can be associated with foraging behaviour in this dataset. Another metric suggesting wider spatial distribution of fish during feeding is that the average CAC value is lower during feeding (0.55 versus 0.59 and 0.61, see Table 3): a more centred distribution is expected when movements are more uniformly distributed.

While the findings align with existing research suggesting that fish distribute themselves more widely and randomly during feeding^69^, the metrics most relevant for measuring the increased area covered by the fish—rel-core-area, rel-home-range, and core-ratio—show minimal variation and differences between the pre- and post-feeding periods compared to during feeding. One explanation may be that effects are obfuscated by the level of unknown variation in data. For instance, the largest effects (although not statistically significant) are due to interactions and account for more than the largest significant factor (Tank). Also, the effect size of residuals is large. As an example of these variations with unknown source is the fact that day 16 distinguishes itself from the other days: in Table 3, almost all metrics are “extreme” compared to the other days often being either minimal or maximal.

### Home range methodology

The proposed metrics and the results from multivariate analysis indicate that home range is a useful tool for monitoring and interpreting the distribution of small numbers of fish over short time scales in experimental tank settings. Specifically, we see that there are systematic differences in spatial distribution between tanks and around feeding events. However, our data set is limited, and the findings need to be corroborated with more data obtained from other systems. Further research should investigate if the metrics are suitable for the real-time monitoring of fish behaviour, for instance by using multivariate statistical control charts which are widely used in the process industry^70^ and is used for monitoring input-based welfare indicators such as temperature^71,72^ in aquaculture. Such systems could potentially be used for the early detection of welfare challenges, or to optimise feeding by continuously assessing appetite-related metrics. Other studies^73–75^ aim to characterize fish distribution within a tank using radial fish distribution, automatically tracked fish, and heatmaps. In an attempt to build up on such research, our methodology allows an observer to not only visualize but to quantify behavioural information as specific group behaviours (e.g., group structure, distance to the wall, most preferred area). Furthermore, this information could be statistically compared at a spatiotemporal level within and/or between tank replicates, thus avoiding a potential subjective bias of individual observers.

### Limitations

The limitations of this study highlight technical challenges for single camera behavioural monitoring such as a lack of depth information, and the impact of camera angle and lighting on keypoint detection. In other settings a stakeholder should consider occlusion in dense fish populations and the influence of water turbidity, which can reduce image clarity and affect detection accuracy. While the lack of depth information does not affect the current findings, incorporating such advancements with the help of stereo cameras would enable a more comprehensive assessment of environmental factors influencing the spatial distribution of fish^13,49,50^. While the number of fish in each group in our study is small, the findings provide valuable insights into the spatial distribution, grouping behaviour, space utilization, and potential welfare issues of Atlantic salmon parr under experimental conditions. Home range and core area metrics can identify underutilized or preferred tank areas, potentially highlighting welfare issues such as suboptimal oxygen levels, temperature gradients, or other environmental factors. This information can guide improvements in tank design to promote better space utilization and enhance fish welfare. In terms of statistical significance related to our results, the main consequence was limited power. In a future application, increasing the number of observations (e.g. days of observation) would enhance this aspect of the design and potentially allow for stronger conclusions. Methodological limitations include the reliance on deep learning models and the inherent complexities in interpreting home range metrics. Also, the concept of home range may not be directly applicable in densely populated tanks where fish are evenly distributed. Additionally, although the statistical analysis, particularly the ASCA, provided valuable insights, the underlying factors driving tank-specific differences in fish behaviour remain unclear due to the lack in data availability. However, with better quality video frames, availability of long-duration video footage from complex scenarios, and advancements in deep learning models, we should be able to enhance the tool to handle different experimental and applied scenarios. Finally, we acknowledge the study’s limited duration and reiterate our focus on demonstrating the tool’s utility for analysing intra- and inter-day behavioural variability, without making claims about longer-term temporal trends.

### Future considerations

In this study, we used a limited number of experimental videos with low numbers of fish as a case study to test our two approaches for documenting the spatial distribution of fish in experimental tank settings. We believe both the tool and the spatial distribution metrics have utility in experimental settings that use similar numbers of fish. By incorporating more training data, our deep learning model can be extended to other fish species and potentially scaled up to larger populations, but this requires testing and evaluation for the given experimental design as we do not know where the home range and core area metrics break down in terms of stocking density.

While we acknowledge that scalability can be a challenge with current restrictions, such as computing power and storage capabilities for processing large high-quality videos, we believe this limitation will diminish over time as technology advances. The rapid development of more efficient AI algorithms, coupled with increasingly affordable and powerful hardware, is expected to make AI-driven analysis much more accessible in the near future. To further advance this research, longer-term studies need to be conducted including analysing larger-scale systems to capture more complex behavioural patterns. In other scenarios it would also be beneficial to examine other potential welfare relevant factors such as variabilities in water current or oxygen levels for specific regions within the tanks. Therefore, incorporating these measurements into future studies will be crucial for a more comprehensive understanding of drivers for how fish distribute themselves around a rearing system.

### Conclusion

This study represents an initial exploration of using computer vision and home range analysis techniques to study fish behaviour. Our approach offers a novel combination of deep learning and home range metrics to provide a more detailed understanding of the spatial distribution of salmon in experimental settings in and around feeding events. While our current dataset is limited to a small number of fish in controlled tank environments, it serves as a foundational step towards understanding the potential of these methods. Analysis of home range metrics indicated differences in spatial distribution and foraging patterns across tanks and days. The application of an existing key-point based software toolbox, DeepLabCut that utilises a deep learning-based approach, allowed for the estimation of home range and core area preferences in relation to feeding that would have been difficult and time consuming to obtain via manual monitoring. An additional utility to this approach was also demonstrating the preferences of fish for certain tank regions outside of feeding periods. meaning it may have potential for documenting how tank design, water flows etc. affect fish distribution. This utility also holds promise for helping stakeholders implement the refinement goals of the 3R’s in animal research and delivering upon the objectives of the European Directive 2010/63/EU on the protection of animals used for scientific purposes^9^.

## Data Availability

The datasets generated and/or analysed during the current study are not publicly available due our ongoing strategic research in this area but are available from the corresponding author on reasonable request.

## Code availability

The source code for the tool can be made available upon request.
